# Death of Woman with Peripartum Influenza B Virus Infection and Necrotizing Pneumonia

**DOI:** 10.3201/eid2007.140230

**Published:** 2014-07

**Authors:** Joshua L. Rein, Aaron M. Etra, Jatinbhai J. Patel, Janet L. Stein, Aimee L. Rivers, Hayley B. Gershengorn, Elizabeth Awerbuch, Barry N. Kreiswirth, Sanjana C. Koshy

**Affiliations:** Beth Israel Medical Center, New York, NY, USA (J.L. Rein, A.M. Etra, J.J. Patel, J.L. Stein, A.L. Rivers, H.B. Gershengorn, E. Awerbuch, S.C. Koshy);; New Jersey Medical School–Rutgers, The State University of New Jersey, Newark, New Jersey, USA (B.N. Kreiswirth)

**Keywords:** Influenza, Methicillin-Resistant Staphylococcus aureus, MRSA, Panton-Valentine leukocidin, pneumonia, pregnancy, peripartum, Respiratory Distress Syndrome, antibiotic, antimicrobial drug

**To the Editor:** Pregnant women are at increased risk for severe influenza-related complications (*1*). Bacterial pneumonia with Panton-Valentine leukocidin-producing (PVL) *Staphylococcus aureus* is infrequently described in the literature as occurring concurrently with influenza B virus infection (*2*–*4*). Additionally, only 2 occurrences of peripartum PVL-methicillin-resistant *S. aureus* (MRSA) pneumonia have been described (*5*,*6*). We report a case of influenza B virus and PVL-MRSA co-infection during pregnancy.

In December 2012, a previously healthy pregnant woman, 38 years of age, at 37 weeks’ gestation and in active labor, sought treatment in a New York hospital reporting 2 days of fever, productive cough, shortness of breath, and pleuritic chest pain. Household contacts included children with influenza-like illness. The patient had declined influenza vaccination while receiving prenatal care. On arrival, examination showed that her temporal temperature was 101.6°F, blood pressure was 122/71 mm Hg, pulse was 121 beats per minute, respiratory rate was 40 breaths per minute, and oxygen saturation was 89% on room air; bilateral inspiratory crackles were heard on lung auscultation. Rapid influenza screening of a nasopharyngeal swab sample by using ELISA was negative for influenza A and B viruses. Culture of the patient’s nares was positive for MRSA colonization. Laboratory evaluation showed leukopenia of 1500/μL, and although imaging was limited by the patient’s lead apron, a chest radiograph demonstrated bibasilar opacities (Figure, panel A). 

**Figure:**
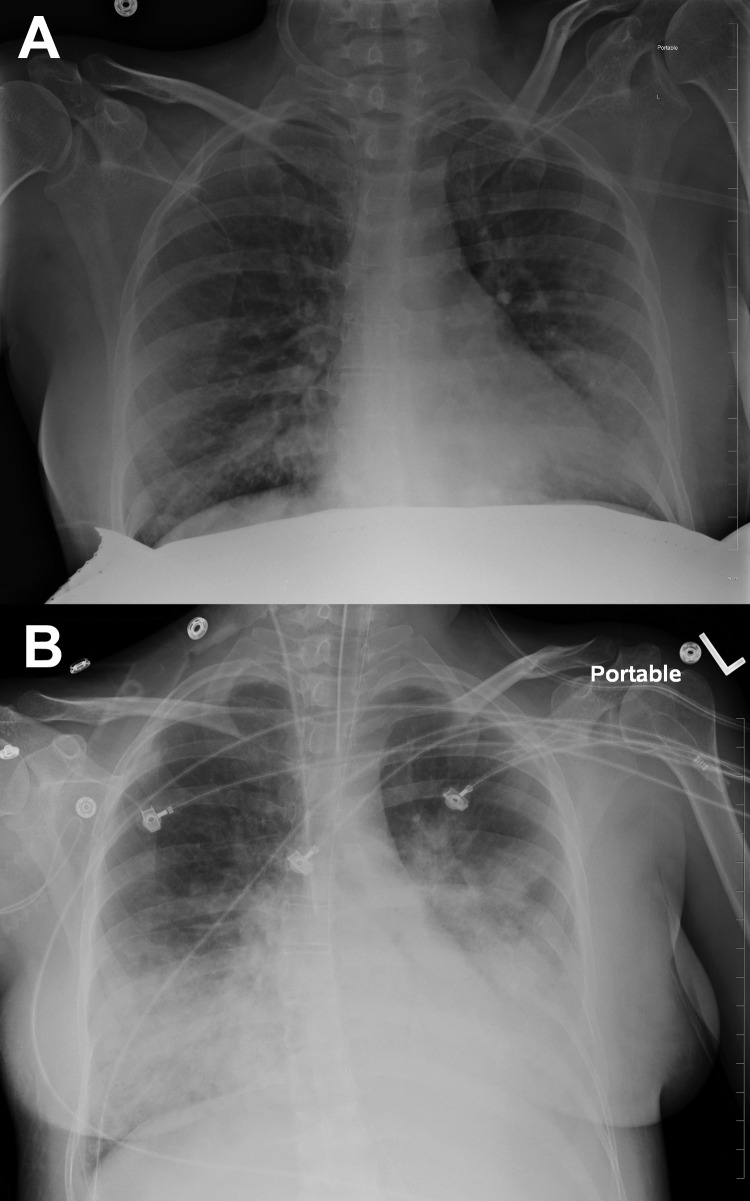
Course of influenza B virus infection and necrotizing pneumonia in peripartum woman, 2012, New York, USA. A) Chest radiograph at time of admission. B) Chest radiograph 1 day later, demonstrating progression of pneumonia.

The differential diagnosis for this patient included influenza pneumonia, community-acquired pneumonia, and MRSA pneumonia; treatment with oseltamivir, ceftriaxone, vancomycin, and azithromycin was started. Because of impending respiratory failure, she was admitted to the Medical Intensive Care Unit where mechanical ventilation was initiated and she underwent a spontaneous vaginal delivery of a live male infant. The patient’s condition deteriorated and progressed to severe acute respiratory distress syndrome with multiple organ failure and required substantial inotropic support. Subsequent laboratory studies showed the following results: leukocyte count 400/μL, lactate 4.2 mmol/L, pH 7.16, PaCO_2_ 36 mm Hg, PaO_2_ 68 mm Hg, HCO_3_ 12 mmol/L, and oxygen saturation of 87% at 1.0 FiO_2_. Repeat imaging demonstrated diffuse infiltrates in all lung fields (Figure, panel B). Because the patient responded poorly to treatment, vancomycin was discontinued and linezolid was started. Despite lung recruitment maneuvers and inhalation of nitric oxide, the patient remained hypoxemic. Extracorporeal membrane oxygenation was initiated and the patient was transferred to another institution. 

After transfer, culture of 1 peripheral blood sample obtained at admission identified MRSA, and viral culture of the patient’s nasal swab sample isolated influenza B virus. Genetic testing of the MRSA isolate identified a PVL-producing USA300 *spa*1 clone carrying staphylococcal cassette chromosome *mec* type IV. The patient died 2 weeks later from overwhelming sepsis. The neonatal course was notable for a birth weight of the infant of 2,825 g and Apgar scores of 5 and 8 at 1 and 5 minutes, respectively. He was intubated and transferred to the Neonatal Intensive Care Unit with an arterial cord blood pH of 6.78 and base deficit of 16 mmol/L. Nasal swab culture isolated methicillin-sensitive *S. aureus*. Viral culture of endotracheal aspirate was negative for influenza A and B viruses. Blood cultures were sterile. He received vancomycin for 1 week and was discharged home to the family on day 8 of life.

This case emphasizes the potential lethality of respiratory complications related to seasonal influenza. Colonization of the patient’s nares with MRSA, possibly PVL-producing, may have predisposed her to a bacterial co-infection, consequentially increasing her risk for death from influenza (*1*). *S. aureus* clones USA300 and USA400 are emerging causes of community-acquired pneumonia in healthy adults and are leading to a rise in co-infections with influenza and MRSA. These 2 infections have been shown to act synergistically in animal models to induce a rapidly progressive necrotizing pneumonia associated with severe leukopenia (*7*). This is unlike classic secondary bacterial pneumonia, which typically occurs in a biphasic course with influenza (*2*).

Although methicillin susceptibility does not influence the mortality rate of PVL-*S. aureus* pneumonia (*8*), antibiotic drugs should be administered early and selection should reflect local resistance patterns. When making the diagnosis, physicians should recognize that the sensitivity of rapid influenza diagnostic tests is low and should not be relied on when a high level of clinical suspicion exists (*1*). Despite trivalent vaccine correspondence with circulating influenza B virus in 5 of 10 influenza seasons during 2001–2011 (*9*), vaccination against seasonal influenza is still the most effective way to prevent this potentially fatal condition. Availability of a quadrivalent influenza vaccination, introduced for the 2013–14 influenza season, should improve future incidence of influenza B virus infection. Because PVL-MRSA colonization is becoming more prevalent (*10*), necrotizing pneumonia must be considered in critically ill patients during influenza season.
